# P-355. Skin Lesions and the Use of Personal Protective Equipment in Healthcare Workers from Lima, Peru during the COVID 19 Pandemic

**DOI:** 10.1093/ofid/ofae631.556

**Published:** 2025-01-29

**Authors:** Carolina Coombes, Paula Arribas-Garcia, Amira Llerena-Delgado, Rodrigo M Carrillo-Larco, Manuel Armando del Solar-Chacaltana

**Affiliations:** Instituto de Medicina Tropical Alexander von Humboldt - UPCH, LIMA, Lima, Peru; Universidad Peruana Cayetano Heredia, Lima, Lima, Peru; Universidad Peruana Cayetano Heredia, Lima, Lima, Peru; Universidad Peruana Cayetano Heredia, Lima, Lima, Peru; Cayetano Heredia Hospital, Lima, Lima, Peru

## Abstract

**Background:**

During the COVID-19 pandemic, healthcare workers relied on personal protective equipment (PPE) to mitigate COVID-19 infections. However, prolonged use of PPE led to a risk of developing skin lesions (SL). In our study, we evaluated the frequency, characteristics and factors associated with SL while using PPE.

**Figure:**
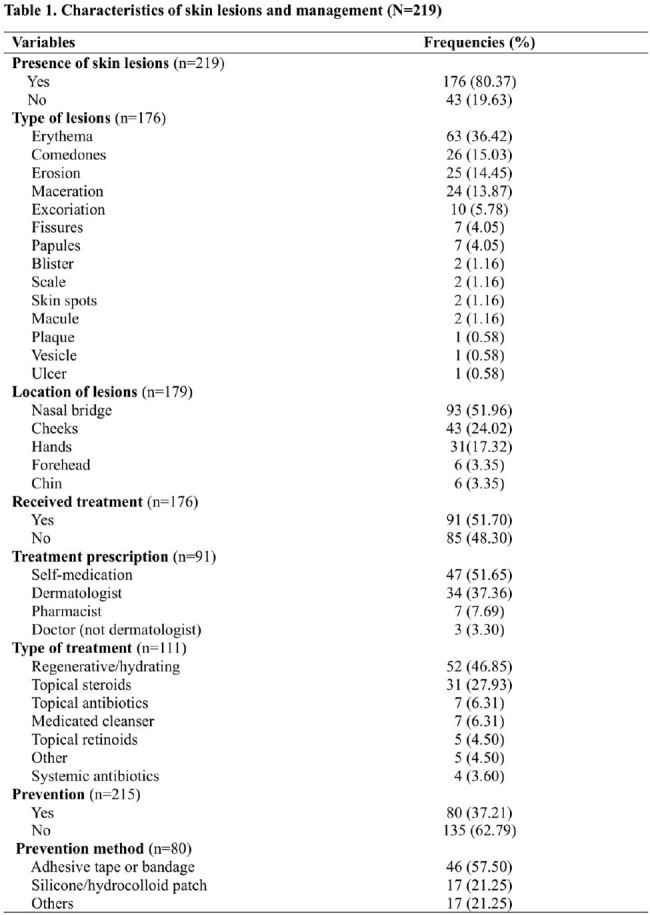

**Methods:**

We conducted a cross-sectional study to survey 219 healthcare workers in two hospitals in Lima, Peru between September 2020 and May 2021. The survey focused on reporting SL related to PPE use during their time as healthcare workers amidst the pandemic. Eligible participants were required to be hospital personnel that was actively working on-site and utilizing PPE.

**Figure:**
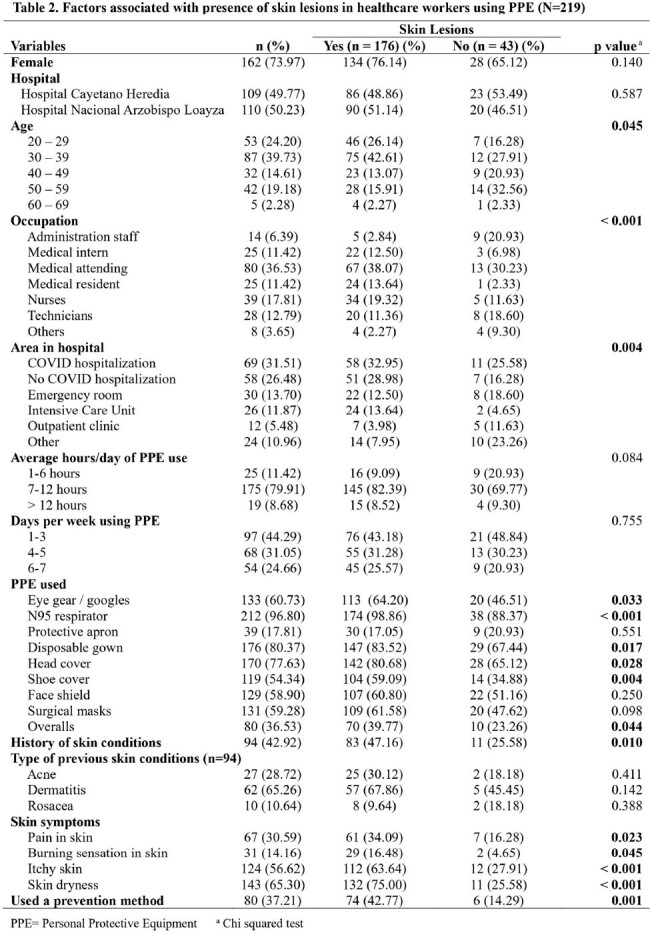

**Results:**

Most commonly used PPE items included N95 respirator (96.80%), disposable gown (80.37%), head cover (77.63%), eye gear (60.73%) and surgical masks (59.28%). 176 participants (80.37%) reported skin lesions while using PPE. The most prevalent SL were erythema (63, 36.42%), comedones (26, 15.03%) and erosions (25, 14.45%). Common locations for SL were the nasal bridge (93, 51.96%) and cheeks (43, 24.02%). Additionally, 91 (51.70%) participants reported seeking treatment, with self-medication (47, 51.65%) being the prevalent method. Among the participants, 80 (37.21%) reported using prevention methods being the most common, adhesive tape or bandages (46, 57.50%). SL were associated with being a medical intern (PR 2.46, p=0.014), medical attending (PR 2.35, p=0.019), medical resident (PR 2.69, p=0.006) and nurse (PR 2.44, p=0.014). Eye gear, shoe covers and overalls were found to be risk factors for SL (PR 1.16, p=0.048; PR 1.21, p=0.007; PR 1.15, p=0.031). Participants who had a history of previous skin conditions were 1.19 (p=0.008) times more likely to have SL. In the adjusted model, working in the ICU (PR 1.31, 95%CI 1.02-1.68, p=0.034), working more than 12 hours per day (PR 1.49, 95%CI 1.07-2.08, p=0.017), presenting a skin burning sensation and skin dryness (PR 1.21, 95%CI 1.03-1.42; p=0.021; PR 1.41, 95%CI 1.04-1.92, p=0.026) were associated risk factors.

**Figure:**
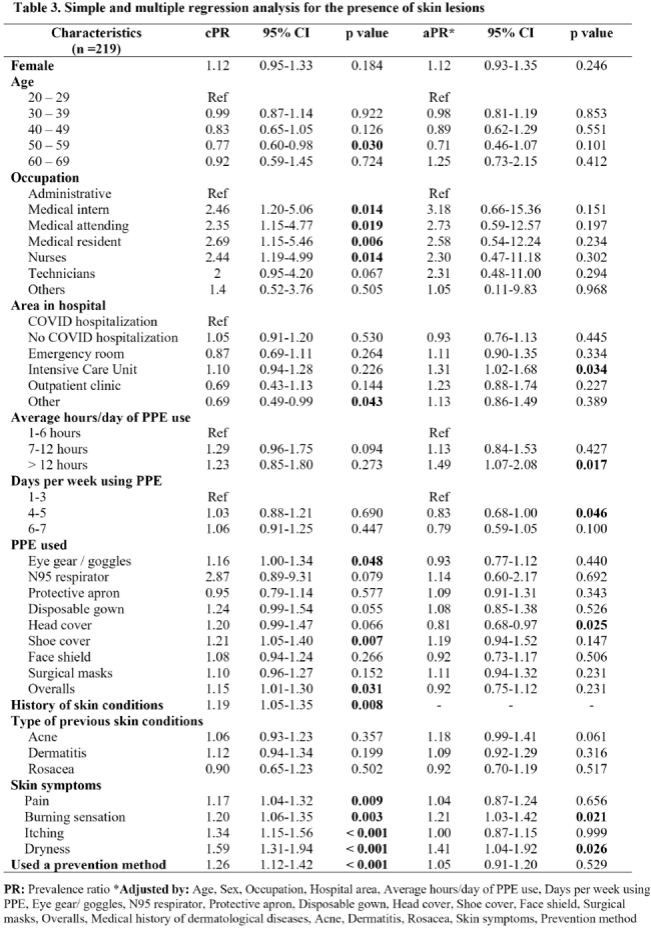

**Conclusion:**

By documenting the prevalence of skin lesions in healthcare worker due to PPE usage we underscore the need for biosafety monitoring, as the presence of lesions can lead to PPE misusage, increasing the risk of respiratory infections.

**Disclosures:**

**All Authors**: No reported disclosures

